# Effects of the intestinal microbiota on prostate cancer treatment by androgen deprivation therapy

**DOI:** 10.15698/mic2022.12.787

**Published:** 2022-11-15

**Authors:** Safae Terrisse, Laurence Zitvogel, Guido Kroemer

**Affiliations:** 1 Medical Oncology, Hôpital Saint-Louis, Paris, France.; 2 INSERM U1015, Equipe Labellisée - Ligue Nationale contre le Cancer, Villejuif, France.; 3 University Paris Saclay, Gif-sur-Yvette, France.; 4 Gustave Roussy, ClinicObiome, Villejuif, France.; 5 Center of Clinical Investigations in Biotherapies of Cancer (CICBT) 1428, Villejuif, France.; 6 Equipe labellisée par la Ligue contre le Cancer, Université de Paris Cité, Sorbonne Université, Institut Universitaire de France, Inserm U1138, Centre de Recherche des Cordeliers, Paris, France.; 7 Metabolomics and Cell Biology Platforms, Gustave Roussy Comprehensive Cancer Institute, Villejuif, France.; 8 Institut du Cancer Paris CARPEM, Department of Biology, Hôpital Européen Georges Pompidou, AP-HP, Paris, France.

**Keywords:** Akkermansia muciniphila, castration-resistant prostate cancer, hormonotherapy, microbiome, Ruminococcus gnavus

## Abstract

Prostate cancer (PC) can be kept in check by androgen deprivation therapy (ADT, usually with the androgen synthesis inhibitor abiraterone acetate or the androgen receptor antagonist such as enzalutamide) until the tumor evolves to castration-resistant prostate cancer (CRPC). The transition of hormone-sensitive PC (HSPC) to CPRC has been explained by cancer cell-intrinsic resistance mechanisms. Recent data indicate that this transition is also marked by cancer cell-extrinsic mechanisms such as the failure of ADT-induced PC immunosurveillance, which depends on the presence of immunostimulatory bacteria in the gut. Moreover, intestinal bacteria that degrade drugs used for ADT, as well as bacteria that produce androgens, can interfere with the efficacy of ADT. Thus, specific bacteria in the gut serve as a source of testosterone, which accelerates prostate cancer progression, and men with CRPC exhibit an increased abundance of such bacteria with androgenic functions. In conclusion, the response of PC to ADT is profoundly influenced by the composition of the microbiota with its immunostimulatory, immunosuppressive and directly ADT-subversive elements.

## INTRODUCTION

The development of different types of cancer is profoundly influenced by the microbiota, which can act locally to directly participate in oncogenesis (e.g., by activating cell-autonomous oncogenic processes or by eliciting pro-carcinogenic chronic inflammation) or affect the evolution of cancers at a distance, mostly by modulating the dialogue between malignant and immune cells in favor of immunosurveillance or its failure [1, 2]. Such long-distance effects are mediated by the intestinal microflora, which is the most abundant and diverse microbiota in the human body. In addition, the composition in the local and intestinal microbiota, as well as its therapy-induced shifts, affect the response of cancers to chemotherapy, targeted therapy and immunotherapy [3, 4]. The connections between the microbiome and prostate cancer from carcinogenesis to response to therapies targeting the androgen pathway are also relevant. Prostate cancer (PC) is not an exception to this general rule, as we will summarize in this minireview.

## CONTRIBUTION OF THE LOCAL MICROBIOTA TO PROSTATE CARCINO-GENESIS AND CANCER PROGRESSION

**Figure 1:**
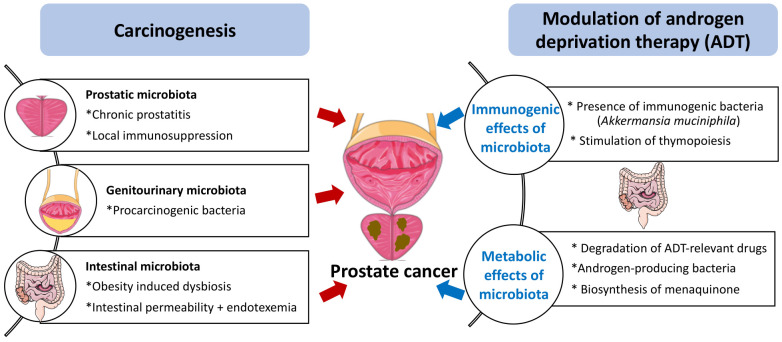
FIGURE 1: Potential mechanisms of action of the microbiota on prostate cancer carcinogenesis and sensitivity to androgen depletion therapy. For details consult text

Benign prostatic hyperplasia and PC are associated with prostatitis, which may be caused by bacteria. Of note, the most prevalent microorganism in the human prostate gland is *Cutibacterium* (formerly known as *Propionibacterium acnes*) [5], which may stimulate the infiltration of the tissue by regulatory T CD4+FoxP3+ cells (Treg) and T helper cell producing interleukin-17 (Th17) cells, hence causing local immunosuppression as well as procarcinogenic inflammation [6]. Thus, the local microbiota present in the genitourinary tract might contribute to carcinogenesis. In this context, it appears interesting that the presence of bacteria (and in particular anaerobic species from 5 genera: *Anaerococcus prevotii, Fenollaria sp. nov., Fusobacterium nucleatum, Porphyromonas sp. nov*. or *asaccharolytica,* and *Peptoniphilus sp. nov*. or *harei*) in urine sediments strongly correlates with poor PC prognosis determined by the D'Amico score [7]. Altogether, the available evidence suggests that the local microbiota might affect prostate cancer progression **(Fig. 1)**. This speculation appears particularly interesting in view of a recent report describing therapeutically relevant immunity against urothelium invasive *Escherichia coli* in the clinical response of bladder cancer patients to immunotherapy with pembrolizumab [8]. However, although *E. coli* is often identified in PC specimens [5], its contribution to procarcinogenic inflammation versus tumor-suppressive immunosurveillance has not yet been investigated.

## CONTRIBUTION OF THE INTESTINAL MICROBIOTA TO PROSTATE CARCINOGENESIS AND CANCER PROGRESSION

Obesity is a major risk factor for PC development and progression [9], as well as a driver of intestinal dysbiosis, which can be defined as an imbalance in the microbes present in the microflora. Dysbiosis is often associated with an increase in gut permeability and endotoxinemia, which is an increase in the circulating level of bacterial lipopolysaccharide (LPS) [10, 11]. In mice, dysbiosis induced by broad-spectrum antibiotics favors the accumulation of LPS in subcutaneous and orthotopic PC, thereby activating local inflammation and promoting tumor growth [12]. High fat diet-induced dysbiosis also triggers an LPS-mediated pro-inflammatory pathway secondary to the LPS-induced upregulation of the histamine-producing enzyme histidine decarboxylase (HDC) in mast cells infiltrating the tumor [13]. Although it is technically feasible to detect LPS in human tissue sections [14], we are not aware of any study investigating the presence of LPS in preneoplastic or malignant prostate specimens. Such studies are urgently awaited. Of note, in PC patients, the fecal abundance of *Proteobacteria* correlates with the presence of distant metastases [12]. Thus, there is circumstantial evidence suggesting that intestinal dysbiosis contributes to prostate carcinogenesis and tumor progression **(Fig. 1)**.

## POSITIVE IMPACT OF THE GUT MICROBIOTA ON THE EFFICACY OF ANDROGEN-DEPRIVATION THERAPY

There are several strategies for the clinical management of PC that range from the absence of immediate action (‘wait and see') to surgical removal of the tumor, as well as from radiation therapy to aggressive chemotherapies [15]. In addition, most PC patients initially respond to androgen deprivation therapy (ADT, usually with the androgen synthesis inhibitor abiraterone acetate or the androgen receptor antagonist such as enzalutamide) before hormone-sensitive PC (HSPC) progresses to castration-resistance PC (CRPC) [16]. Although much emphasis has been laid on the cell autonomous response of PC cells to ADT and the consequent HSPC-CRPC transition, there is evidence that ADT also acts through PC cell non-autonomous mechanisms involving the immune system. Thus, in a mouse model of PC, the therapeutic response to ADT depends on the immune system, as indicated by the fact that antibody-mediate depletion of T cells or genetically determined athymia compromise the ADT-mediated PC control [17]. This appears to be applicable to PC patients because successful (long-term) ADT results in an increase of thymic output, as indicated by the augmentation of recent thymic emigrant cells (i.e., signal joint T-cell receptor excision circles, abbreviated as sjTRECs) in peripheral blood [17].

As true for other cancers treated by immunogenic chemotherapy or immunotherapy [1, 2, 3, 4], the gut microbiota plays a major role in determining the therapy-relevant immune response elicited by ADT against PC. Thus, in the PC mouse model, depletion of the gut microbiota by orally administered broad-spectrum antibiotics reduces the efficacy of ADT. In mice, PC reduces the relative abundance of a particular immunostimulatory bacterium, *Akkermansia muciniphila*, in the gut, and this effect was reversed by ADT. Moreover, cohousing of PC-bearing mice with tumor-free mice or oral gavage with *A. muciniphila* ameliorates the efficacy of ADT. CRPC (but not HSPC) patients manifest a shift in the composition of their fecal microbiota that correlated with sjTRECs [17]. Although these results plead in favor of the beneficial impact of specific bacteria on the ADT-triggered anti-PC response. Nonetheless, the exact role of *A. muciniphila* in human PC remains controversial. For example, *A. muciniphila* might elicit both immune-dependent and immune-independent anticancer effects. Thus, in abiraterone-treated patients progressing towards CRPC, *A. muciniphila* expands, correlating with an increase in the biosynthesis of menaquinone (vitamin K2) [18] which inhibits PC growth in vitro, i.e., in an immune-independent fashion [19]. Conversely, extracellular vesicles derived from *A. muciniphila* can elicit cytotoxic T lymphocyte responses against PC in mice [20]. Moreover, according to one report, the clinical response to immunotherapy by PD-1 blockade of metastatic CRPC progressing on enzalutamide is associated with a decrease rather than with an increase in *A. muciniphila* [21], contrasting with the observation that *A. muciniphila* is associated with clinical responses to PD-1 blockade in other human malignancies including non-small cell lung cancer, melanoma and uroepithelial cancer [4, 22, 23]. Instead, the response of CRPC patients to PD-1 blockade correlated with an increase in the fecal abundance the *Streptococcus salivarius* [21]. Thus, different bacteria other than *A. muciniphila* may contribute to the clinical response of PC patients to immunotherapy. Irrespective of this detail, it appears clear that the gut microbiota contributes to the efficacy of ADT against PC **(Fig. 1)**.

## NEGATIVE EFFECTS OF THE GUT MICROBIOTA ON THE EFFICACY OF ANDROGEN-DEPRIVATION THERAPY

In sharp contrast with the afore-mentioned positive effects of the gut microbiota on ADT responses, there is also evidence supporting a deleterious role for specific bacteria in the therapeutic response. Thus, ADT of prostate cancer patients reportedly causes a decrease in α and β-diversity of the gut microbiota, which might precede or accompany the development of dysbiosis [24]. Prior reports indicate that specific bacteria contained in human feces can regulate androgen metabolism [25, 26, 27, 28]. More importantly, however, it appears that bacteria can interfere with the pharmacokinetics and pharmacodynamics of ADT-relevant drugs.

On one hand, abiraterone can be metabolized by the gut microbiota in mice [29]. However, systematic clinical studies of the impact of intestinal bacteria on the half-life of abiraterone or other ADT-targeting molecules are elusive. On the other hand, perhaps more importantly, the gut microbiota from patients with CRPC or castrated mice can convert androgen precursors into active androgens, which are absorbed into the systemic circulation [30]. When the gut microbiota is depleted in mice, circulating DHEA and testosterone levels are significantly reduced. Furthermore, the *Ruminococcus* and *Bacteroides* genera are increased in the gut microbiota of CRPC patients compared to HSPC patients. In patients with CRPC, the *Ruminococcus* genus is associated with poor prognosis, while *Prevotella* is associated with favorable prognosis. *Ruminococcus gnavus* and *Bacteroides acidifaciens* can convert pregnenolone and hydroxypregnenolone into androgens including DHEA and testosterone, and oral gavage with *R. gnavus* can indeed accelerate PC growth upon surgical castration. In contrast, the abundance of androgens is reduced and tumor growth is controlled upon fecal microbiota transfer (FMT) from hormone-sensitive prostate cancer patients or the administration of *Prevotella stercorea* into mice [30]. The mechanism through which *P. stercorea* reduces androgen production by the intestinal microbiota remains to be determined. Apart from this uncertainty, the available data plead in favor of the possibility that an androgen-producing microbiota subverts the impact of ADT against PC **(Fig. 1)**.

## CONCLUSION

As summarized in this review, the pathogenesis of PC, which - as true for other malignant diseases - involves chronic inflammation as well as failing immunosurveillance, may be conditioned by local infection and pro-inflammatory microbial products such as LPS. Intriguingly, it appears that the gut microbiota plays a decisive role in determining the fate of PC patients under ADT. Since ADT, at least in part, must induce an anticancer immune response to be efficient and the immune system is under the tonic influence of the intestinal microflora, dysbiosis and depletion of immunostimulatory bacteria may have a negative impact on the therapeutic efficacy of ADT. In addition, bacteria in the gut affect the metabolism of ADT-relevant drugs, reducing their concentrations in circulation, and produce steroids that interfere with hormone therapies (**Fig. 1**).

## Abbreviations:

PC: – prostate cancer,
CRPC: – castration-resistant prostate cancer,
Treg: – T CD4+FoxP3+ cells,
Th17: – interleukin-17,
LPS: – lipopolysaccharide,
HDC: – histidine decarboxylase,
ADT: – androgen deprivation therapy,
HSPC: – hormone-sensitive PC,
CRPC: – castration-resistance PC,
sjTRECs: – signal joint T-cell receptor excision circles,
FMT: – fecal microbiota transfer.
